# Genome Sequences of Six Wheat-Infecting *Fusarium* Species Isolates

**DOI:** 10.1128/genomeA.00670-13

**Published:** 2013-09-05

**Authors:** Paula M. Moolhuijzen, John M. Manners, Stephen A. Wilcox, Matthew I. Bellgard, Donald M. Gardiner

**Affiliations:** Centre for Comparative Genomics, Murdoch University, Perth, Australiaa; Commonwealth Scientific and Industrial Research Organisation (CSIRO) Plant Industry, Black Mountain, Canberra, Australian Capital Territory, Australiab; Queensland Bioscience Precinct, Brisbane, Queensland, Australiad; Australian Genome Research Facility, the Walter and Eliza Hall Institute of Medical Research, Parkville, Victoria, Australiac

## Abstract

*Fusarium* pathogens represent a major constraint to wheat and barley production worldwide. To facilitate future comparative studies of *Fusarium* species that are pathogenic to wheat, the genome sequences of four *Fusarium pseudograminearum* isolates, a single *Fusarium acuminatum* isolate, and an organism from the *Fusarium incarnatum-F. equiseti* species complex are reported.

## GENOME ANNOUNCEMENT

Diseases incited by *Fusarium* pathogens are among the major biotic constraints on wheat and barley crop production (1). Furthermore, many cereal-infecting fusaria produce mycotoxins that are hazardous to humans and animals. *Fusarium* pathogens can cause three diseases on cereals (head blight, crown rot, and root rot), depending on the host developmental stage. Head blight disease causes enormous losses in northern hemisphere production areas, while crown rot is prevalent in arid regions. In Australia, crown rot is responsible for annual losses of ~$100 million in the wheat and barley industries (2, 3). In drier wheat-growing regions, *Fusarium pseudograminearum*, causing crown rot disease, predominates (4–6) and is the most commonly identified species in Australian wheat fields. The *Fusarium incarnatum-F. equiseti* species complex (FIESC) and *Fusarium acuminatum* isolates, which are less often associated with major disease epidemics, are regularly identified as co-occurring with the other pathogens in field surveys (4, 7). To undertake comparative analyses, six separate genomes covering three *Fusarium* species were sequenced.

Isolates from the CSIRO Plant Industry *Fusarium* collection (Brisbane, Australia) were selected for sequencing after confirming species identifications by sequencing the elongation factor 1 alpha gene (8). Four *F. pseudograminearum* isolates were chosen (Table 1) from different provenances to augment the already existing *F. pseudograminearum* genome (9). Single isolates of *F. acuminatum* and a FIESC member (10) confirmed as species 5 (K. O’Donnell, personal communication) were also sequenced (Table 1). All isolates were tested for virulence on wheat using a laboratory assay for crown rot infection (9).

**TABLE 1:** Isolate provenance, genome assembly, and annotation statistics

Species	Isolate	Locus	Isolate provenance (Australia)	No. of scaffolds	No. of N_50_^*a*^	N_50_^*a*^ (Mbp)	Max^*a*^ (Mbp)	Sum (Mbp)	No. of coding genes	Accession no.
*Fusarium* sp. FIESC5	CS3069	BN850	Wilga Downs, Queensland	3,555	321	0.03	0.25	38.02	13,743	CBMI010000000
*F. pseudograminearum*	CS3220	BN846	Liverpool plains, New South Wales	191	12	1.07	2.7	37.16	12,615	CBMC010000000
*F. pseudograminearum*	CS3427	BN847	Wilga Downs, Queensland	182	12	0.93	2.02	37.07	12,577	CBMD010000000
*F. pseudograminearum*	CS3487	BN848	Tamworth, New South Wales	364	20	0.62	1.49	37.01	12,749	CBME010000000
*F. pseudograminearum*	CS5834	BN849	Tammin, Western Australia	228	12	1.04	2.51	37.48	12,633	CBMF010000000
*F. acuminatum*	CS5907	BN851	Stockdale, Western Australia	716	79	0.17	0.61	43.89	15,353	CBMG01000000

a N_50_ and Max statistics refer to scaffold sequences.

Illumina TruSeq DNA sequencing libraries were constructed from *in vitro*-grown mycelium-derived DNA. The average insert size range was 383 to 494 bp. Bar-coded samples were sequenced with HiSeq 2000 100-bp paired-end reads. In Yabi (11), the read sequences were assessed for quality with FastQC v0.10.0 (12). Poor quality sequence, adaptors, and redundant reads were removed with ConDeTri v2.0 (13) and rmDupPCR.pl (13), respectively. Any contaminating foreign DNA was filtered out using BWA v0.6.1 (14), samTools v0.1.18 (15), NCBI bacterial genomes, and International Wheat Genetics Symposium (IWGS) wheat survey sequences (16). Mitochondrial sequence was filtered from chromosomal data using BWA alignment to NCBI mitochondrial genomes. The genome and mitochondria were assembled with Velvet v1.2.03 (17) at an optimal hash length of 53. Assembly statistics are given in Table 1. Scaffolds were mapped at a minimum 70 percent identity to the *Fusarium graminearum* PH-1 genome (accession no. CM000575.1) (18) using BLAT v34 (19) into a pseudochromosomal sequence. Low complexity and repeat elements were masked with the RepeatMasker v3.3.0 (20) fungi repeat library and Censor v4.2.28 (21) *Fusarium* library. Protein-coding gene predictions were made with GeneMark-ES v2.3e (22) and Augustus v2.5.5 (23), and *F. pseudograminearum* CS3096 Illumina RNA-Seq unstranded reads were assembled with TopHat v1.4.0 (24) and CuffLinks v1.3.0 (25). Noncoding RNAs were predicted using Aragorn v1.2.33 (26) and RNAmmer v1.2 (27). Protein-coding genes were annotated with AutoFACT v3.4 (28). While most of the genomes encoded around 13,000 predicted proteins, the *F. acuminatum* genome was predicted to encode 15,353 proteins. This genome was also the largest, at 44 Mbp. Sequence data, including scaffolds and pseudochromosomal scaffolds, have been deposited in EMBL.

### Nucleotide sequence accession numbers.

The six whole-genome shotgun projects have been deposited at DDBJ/EMBL/GenBank under umbrella BioProject no. PRJEB1746. The whole-genome sequence (WGS) accession numbers can be found in Table 1. The versions described in this paper are the first versions.
